# On the History of dermatology: the study of skin diseases over the centuries - Response to the observations and comments by Mirzaei MR et al. and Rashmi TM et al^☆^^☆☆^

**DOI:** 10.1016/j.abd.2021.07.001

**Published:** 2021-07-22

**Authors:** Iago Gonçalves Ferreira, Magda Blessmann Weber, Renan Rangel Bonamigo

**Affiliations:** a Universidade Federal de Ciências da Saúde de Porto Alegre, Porto Alegre, RS, Brazil; b Santa Casa de Misericórdia de Porto Alegre, Porto Alegre, RS, Brazil; c Faculty of Medicine, Universidade Federal do Rio Grande do Sul, Porto Alegre, RS, Brazil

Dear editor,

We are grateful for the comments of “Earliest details of Dermatology by Ayurveda”^1^ on our publication “History of dermatology: the study of skin diseases over the centuries”.^2^ The information provided by the letter is interesting and complementary in one aspect, which demonstrates the rich history of dermatology throughout the centuries and in the most varied regions of the world.

Similarly, we are grateful for the comments by Mirzaei MR et al.^3^ regarding our publication. The information provided is correct and is an addition to the published information. The history of Dermatology is complex, very rich and there are always very important data to be informed.

## Financial support

None declared.

## Authors’ contributions

Iago Gonçalves Ferreira: Conception and/or design of the study; literature review and article selection; content analysis; results analysis; preliminary review and final drafting.

Magda Blessmann Weber: Conception and/or design of the study; literature review and article selection; content analysis; preliminary review and final drafting.

Renan Rangel Bonamigo: Conception and/or design of the study; literature review and article selection; content analysis; preliminary review and final drafting.

## Conflicts of interest

None declared.
